# Nuanced Gender Differences in Everyday Ageism and Health

**DOI:** 10.1093/geroni/igaf122.100

**Published:** 2025-12-31

**Authors:** Julie Ober Allen

**Affiliations:** University of Wisconsin–Madison, Madison, Wisconsin, United States

## Abstract

Qualitative research has documented substantial gender differences in perceptions, experiences, and consequences of ageism, yet survey studies have generally documented comparable amounts of self-reported ageism for men and women. This study uses the new Everyday Ageism Scale for an in-depth investigation of gender differences in amount of routine ageism experienced, types, and salience to mental and physical health in a nationally representative sample of US adults ages 50-80 (2019 National Poll on Healthy Aging, N = 2044). Men and women reported comparable amounts of everyday ageism overall and two of three types (age discrimination, internalized ageism). Women reported more exposure to ageist messages suggesting aging and older adults were unattractive, undesirable, and a source of ridicule (p=.005). No gender differences were detected in associations between 1) overall amount of ageism and all examined mental and physical health outcomes, and 2) types of ageism and physical health outcomes (number of chronic health conditions, odds of fair/poor physical health). Among types of ageism, age discrimination and internalized ageism were associated with mental health outcomes at comparable levels by gender; however, more exposure to ageist messages was associated with higher odds of having depression symptoms among women (unrelated for men) and lower odds of fair/poor mental health among men (unrelated for women). While ageism may be a health risk shared by men and women, findings inform initiatives and clinical care on nuances at the intersection of ageism and gender that may contribute to differences in types of ageism experienced and potential mechanisms affecting mental health.

